# XAB06, a glyco-humanized broad-spectrum anti-*Pseudomonas aeruginosa* polyclonal antibody, protects mice in a lethal sepsis model

**DOI:** 10.1128/aac.00277-26

**Published:** 2026-09-03

**Authors:** Pierre-Joseph Royer, George Graur, Mehdi Varin, Bernard Vanhove, Odile Duvaux, Firas Bassissi

**Affiliations:** 1 Xenothera, Nantes, France; Columbia University Irving Medical Center, New York, New York, USA

**Keywords:** glyco-humanized polyclonal antibody, *Pseudomonas aeruginosa*

## Abstract

The global rise of antibiotic-resistant *Pseudomonas aeruginosa* (PA) necessitates the development of novel therapeutic approaches. Polyclonal antibodies constitute a promising approach, as they target multiple bacterial epitopes and functions. Moreover, they have recently demonstrated renewed clinical applicability in their humanized format. We developed a glyco-humanized polyclonal antibody (GH-pAb) targeting PA by immunization of pigs with five PA serotypes frequently encountered in clinical settings. This pentavalent GH-pAb, named XAB06, exhibited binding activity against the five serotypes used for the immunization, as well as unrelated PA serotypes. No binding activity was observed against *Escherichia coli, Klebsiella pneumoniae, Enterobacter cloacae,* or *Acinetobacter baumannii*, demonstrating the specificity of XAB06 against PA. In addition, XAB06 showed no reactivity against human blood cells. Functional assays revealed that XAB06 significantly inhibited swarming motility, biofilm formation, and pyocyanin production, indicating interference with key virulence determinants of PA. Notably, XAB06 conferred protection in a murine model of sepsis using the reference strains PAO1, PA103, and a ST235 high-risk clone. Collectively, these findings demonstrate the broad PA coverage and the protective activity of XAB06, supporting its potential as a novel therapeutic strategy against PA.

## INTRODUCTION

*Pseudomonas aeruginosa* (PA) is a gram-negative bacterium with a ubiquitous distribution in natural settings. PA is an opportunistic and very versatile pathogen that can cause local (pulmonary, urinary, and gastrointestinal tract) or systemic (bacteremia) and acute (indwelling medical devices) or chronic (infection in cystic fibrosis patients) infections. PA is a major threat in hospitals where it represents 10%–15% of the nosocomial infections (1–3). It is a member of the ESKAPE group, comprising life-threatening antibiotic-resistant bacterial pathogens. These features led PA to be ranked as a high-priority pathogen in 2024 by the World Health Organization (4).

Pathogenicity of PA arises from a remarkable capacity for adaptation leading to the development of intrinsic or extrinsic resistance mechanisms. In 2021, in the EU/EEA, 31% of the PA isolates reported to the European antimicrobial resistance surveillance network were resistant to at least one antimicrobial drug, and 4% were multidrug resistant (MDR), i.e., resistant to three or more classes of antimicrobial drugs (5). In the United States, the overall prevalence of healthcare-associated MDR-resistant isolates reached 7.7% in 2023 (6). Particularly vulnerable are patients in intensive care units or patients with bloodstream infections, with a prevalence of multidrug resistance isolates around 20% and a mortality rate ranging from 30% to 60% (7, 8). In addition, PA produces an abundance of virulence factors, non-exhaustively including membrane components such as lipopolysaccharide (LPS), which is involved in resistance to phagocytosis and serum killing; flagellin (motility); and type V pili (host-cell attachment), as well as secreted factors such as toxins (cell death induction and suppression of immune response), proteases (degradation of host tissues), or exopolysaccharides (biofilm formation) (3, 9). Production of virulence factors and resistance to antibiotics are finely tuned at the population level by the three quorum-sensing networks, allowing cell-to-cell communication between isolated individuals (10, 11).

New therapeutic strategies are thus desperately needed in the fight against PA, especially with an antibiotic pipeline that remains insufficient and mainly focused on upgrading existing antibiotics (8). Recent years have seen the use of antibodies (Ab) in the field of infectious disease (12, 13). Promising preclinical results against PA have been obtained with monoclonal antibodies (mAbs) targeting the type 3 secretory system (T3SS) virulence factor (14–16), flagellin (17, 18), or the pyoverdine receptor (19). A bispecific mAb (MEDI3902, Gremubamab) directed against the membrane T3SS and the secreted PSL exopolysaccharide went up to phase II, with mitigated results in PA-colonized ventilated patients (20–23). Its therapeutic potential is currently evaluated in patients with bronchiectasis (20). Nevertheless, mAbs are limited by their single target mode of action, allowing immune escape mechanisms, their costs, and their manufacturing difficulties (24). As they target a wide array of antigens, polyclonal Abs (pAbs) offer broad neutralization coverage and are expected to minimize the risk of immune evasion and antimicrobial resistance (25, 26). Polyclonal Abs can also be deployed rapidly in case of emergency situations, as shown during the recent SARS-CoV-2 pandemic (27). The heterogeneous nature of pAbs poses specific regulatory challenges, and batch-to-batch consistency is a critical point in the development of pAbs. It is controlled by quality-by-design principles, orthogonal analytical methods, and standardized potency assays. Today, more than 20 pAbs have been approved by the Food and Drug Administration or the European Medicines Agency (24). Yet, the clinical development of pAbs remains hindered by their immunogenicity due to the presence of xenogeneic patterns, mainly N-glycolylneuraminic acid (Neu5Gc) and α1,3 galactose (α-Gal) (28). The production of glyco-humanized (GH)-pAbs, devoid of those xenogeneic carbohydrate patterns, offers new perspectives for the therapeutic use of pAbs. GH-pAbs have shown promising therapeutic proficiency and a satisfactory safety profile in clinical settings, in SARS-CoV-2 disease (NCT04453384 and NCT04928430) (25), or in the setting of solid organ transplantation (NCT04431219) (29).

The International Antigenic Typing Scheme identified 20 serotypes of PA (O1–O20) based on the O-polysaccharide structure, the outermost portion of LPS on the bacterial surface (30). Serotype distribution of clinical PA isolates is not homogeneous, and serotypes O1, O2, O4, O6, and O11 account for more than 60% of infections (31–34). Here, we present the production and characterization of XAB06, a GH-pAb raised against a combination of O1, O2, O4, O6, and O11 PA serotypes. Activity of XAB06 against PA was investigated *in vitro*, and protective efficacy was assessed *in vivo* in a mouse sepsis model.

## MATERIALS AND METHODS

### Bacterial strains

The following PA strains were obtained from the Collection de l’Institut Pasteur (CIP, Paris, France): CIP 59.33 (ATCC 33348, serotype O1); CIP 59.34 (ATCC 33349, O2); CIP 59.35 (ATCC 33350, O3); CIP 59.36 (ATCC 33351, O4); CIP 59.37 (ATCC 33352, O5); CIP 59.39 (ATCC 33354, O6); CIP 59.41 (ATCC 33356, O9); CIP 59.43 (ATCC 33357, O10); CIP 59.44 (ATCC 33358, O11), CIP 104116 (ATCC 15692, PAO1), and CIP102967 (ATCC 29260, PA103). High-risk ST235 clone (H27244 [35]) was from Smaltis (Besançon, France). *Escherichia coli* (*EC25922*)*, Klebsiella pneumoniae* (*KP13883*)*, Enterobacter cloacae* (*Ecl13047*), or *Acinetobacter baumannii* (*AB19606*) were kindly provided by Cédric Jacqueline (Nantes Université). All strains were stored at −80°C in LB medium containing 15% glycerol (Sigma-Aldrich).

### Preparation of *Pseudomonas* vaccine and pig immunization

Bacteria from frozen stock were grown overnight at 37°C on tryptic soy agar plates (Sigma-Aldrich, Saint-Quentin-Fallavier, France). Subcultures were then performed and maintained at 37°C for 36 h on tryptic soy agar. Bacteria were collected in PBS and heat inactivated in a microbiology incubator (Memmert, Eagle, WI, USA). PA inactivation was confirmed by sterility tests according to the European Pharmacopeia (Carso Pharma, Venissieux, France). Pigs double knocked out for cytidine monophospho-N-acetylneuraminic hydroxylase and alpha-1,3-galactosyltransferase (GGTA1), the two enzymes responsible for xenogeneic glycosylation, were immunized with inactivated PA. Blood was collected from the fourth immunization from the jugular vein after sedation with 2 mg azaperone/kg of body weight (Elanco, Sevres, France). Blood was stored at room temperature for 3–5 h. Serum was separated from the clot by centrifugation, and IgG was purified with an AKTA Prime chromatography system (Cytiva, Saint-Germain-en-Laye, France) using a ProSep Ultra Plus Protein A resin (Merck, Molsheim, France).

### PcrV and whole-bacterium ELISA

For the PcrV ELISA, recombinant PcrV (AntibodySystem, Paris, France) at 1 µg/mL in carbonate/bicarbonate buffer was immobilized on high-binding ELISA plates (Nest Scientific Inc., Woodbridge, NJ, USA) by overnight incubation at 4°C. For whole-cell ELISA, bacteria were grown on tryptic soy agar plates for 36 h before heat inactivation. Then, 100 µL of inactivated bacteria (approximately 4 × 10^8^ CFU/mL, estimated from OD_600_ measurement, considering that an OD_600_ of 1 corresponds approximately to 10^9^ cells/mL) was plated and left to dry for 72 h under a running biosafety cabinet. Wells were then washed with PBS containing 0.05% Tween 20 and saturated for 1 h at room temperature with PBS containing 0.05% Tween 20 and 5% skimmed milk. Serum, XAB06, non-immune control IgG, or Gremubamab (MedChemExpress, Monmouth Junction, NJ, USA) (100 µL in PBS/Tween/milk) were added for 2 h at room temperature. After three washes with PBS/Tween, bound antibodies were detected with 100 µL of HRP-conjugated protein A (Sigma-Aldrich) in PBS/Tween/milk for 1 h at room temperature. Binding was revealed by the addition of 100 µL of TMB (Sigma-Aldrich). The reaction was stopped with 50 µL of 1 M H_2_SO_4_, and OD was measured at 450 nm (with a reference at 600 nm) with a microplate reader (Tecan, Männedorf, Switzerland).

### C1q recruitment assay

ELISA plates were prepared as for the whole bacteria ELISA assay. XAB06 or non-immune control IgG in PBS containing 0.05% Tween 20 and 5% skimmed milk was added for 1 h at RT to inactivated bacteria-coated ELISA plates. After three washes with PBS/Tween, human C1q (Sigma-Aldrich) (2 µg/mL in PBS/Tween/milk) was added for 1 h at RT. Bound C1q was then detected with a sheep FITC-anti-human C1q, followed by a rabbit HRP-anti-FITC (both from Bio-Rad, Marnes-La-Coquette, France). ELISA was developed by adding 100 µL of TMB. The reaction was stopped with 50 µL of 1 M H_2_SO_4_, and OD was measured at 450 nm (with a reference at 600 nm).

### Immunofluorescence staining

Microbial samples were spotted onto microscope slides, air-dried, and covered for 2 min with ice-cold 100% methanol. Samples were saturated for 1 h at room temperature with PBS containing 0.05% Tween 20 and 5% skimmed milk. Slides were incubated for 1 h at room temperature with XAB06 or non-immune control IgG at 10 µg/mL in PBS/Tween/milk. After three washes with PBS/Tween/milk, bound XAB06 was detected with AF488-conjugated goat anti-swine IgG (Jackson ImmunoResearch, Cambridgeshire, UK). After washing, samples were stained with DAPI (1 µg/mL) and mounted in ProLong Gold solution (Thermo Fisher Scientific, Waltham, MA, USA). Images were acquired on an Eclipse Ts2 microscope with NIS-Elements software (Nikon, Champigny-sur-Marne, France).

### Reactivity with human blood cells

Human peripheral blood mononuclear cells (PBMCs) from healthy donors were obtained from Etablissement Français du Sang (Nantes, France). For binding, 100,000 PBMCs in 100 µL of PBS containing 2 mM EDTA and 0.5% bovine serum albumin (BSA) (both from Sigma-Aldrich) were incubated with XAB06 or anti-lymphocyte IgG (maximum concentration, 1,000 µg/mL, followed by 1:3 dilution) for 1 h at 4°C. After washing with PBS/EDTA/BSA, detection of bound IgG was performed with AF488-conjugated goat anti-swine IgG. After washing with PBS/EDTA/BSA, fluorescence was measured on a NucleoCounter NC-300 Advanced Image Cytometer (Chemometec, Allerød, Denmark).

For complement-dependent cytotoxicity, human PBMCs were incubated with increasing concentrations of IgG in the presence of rabbit complement (1/6) (Cedarlane Burlington, Canada). After 90-min incubation at 37°C and 5% CO_2_, cells were washed, and viability was measured using the CellTiter-Glo assay (Promega, Charbonnières-Les-Bains, France) on a GloMax Navigator microplate luminometer (Promega).

For the cytokine secretion assay, 100,000 PBMCs in 100 µL of RPMI medium with 10% fetal calf serum (Biowest, Nuaillé, France) and 1% penicillin/streptomycin (Sigma-Aldrich) were seeded into 96-well U-bottom plates (Corning, Boulogne-Billancourt, France). PBMCs were treated with XAB06 or a combination of phorbol 12-myristate 13-acetate (PMA; 50 ng/mL) and ionomycin (1 µg/mL) (both from Sigma-Aldrich). After 24 h culture at 37°C and 5% CO_2_, cell supernatants were collected, and concentrations of interleukin-6 (IL-6) and tumor necrosis factor α (TNFα) were determined with human OptEIA ELISA sets (BD Biosciences, Le Pont de Claix, France) according to the manufacturer’s instructions.

### Biofilm quantification

Bacterial suspensions of PA (100 µL, 80 × 10^6^ CFU/mL in tryptic soy broth) were cultured in the presence or absence of XAB06 in 96-well flat-bottom plates at 37°C under slow agitation (30 rotations per minute). Biofilm was quantified after 6 h to avoid dispersal that can occur with longer incubation periods (36). Biofilm was quantified based on a previous method with modifications (37). Briefly, plates were washed four times with distilled water, and biofilm was stained with 0.5% crystal violet (Sigma-Aldrich) for 25 min at room temperature. Plates were then washed four times with distilled water. Glacial acetic acid was finally added to disrupt the biofilm and solubilize the crystal violet. Biofilm was quantified by measuring OD at 600 nm on a microplate reader.

### Bacterial swarming motility

The bacterial motility was investigated on semi-solid 0.7% agar plates according to a method described by Köhler et al. (38) with modifications. Briefly, tryptic soy agar was heated until melting, then cooled to 45°C–50°C, and XAB06 was added to the medium before pouring into plates. PA were grown under agitation for 3 h at 37°C in tryptic soy broth. A suspension of PA (4 µL, 200 × 10^6^ CFU/mL) was then deposited onto agar and allowed to dry. The bacterial motility area was then measured after 24 and 48 h of culture at 37°C.

### Quantification of pyocyanin production

PA were grown under agitation for 3 h at 37°C in tryptic soy broth. Bacterial suspensions of PA (10^6^ CFU/mL) were then prepared in tryptic soy broth and cultured in 12-well plates for 24 h at 37°C under slow agitation (30 rotations per minute). Cultures (2 mL) were then centrifuged at 4,200 rpm for 30 min. Supernatants were collected, and 1 mL of chloroform was added. After thorough vortexing (3 × 10 s, 5 min incubation in between), samples were centrifuged at 4,200 rpm for 10 min. Supernatants were discarded, and 0.375 mL of 0.2 M hydrochloric acid was added to the lower, blue-colored chloroform phase. After 30 min of incubation with intermittent vortexing, samples were centrifuged at 4,200 rpm for 10 min. The resulting pink supernatants were collected, and OD at 520 nm was measured. The pyocyanin concentration was calculated using the following formula: pyocyanin content (µg/mL) = OD_520_ × 17.072, where 17.072 represents the molar extinction coefficient of pyocyanin at 520 nm (39).

### Mouse sepsis model

Pathogen-free RjOrl:SWISS female mice (8- to 12-week-old) (Janvier-Labs, Le-Genest-Saint-Isle, France) were maintained at the UTE-IRS2 Nantes Biotech Animal Facility, with free access to food and water. To select the infectious dose, serial dilutions of broth cultures in 5% porcine gastric mucin (Sigma-Aldrich) were injected intraperitoneally into mice. Porcine gastric mucin enhances the infectivity of pathogens and allows the bacterial load to be reduced (40). The precise inoculum size was determined by dilution plating. The lowest dilution at which all the animals died within 24–48 h was defined as the minimal lethal dose (LD100). For the infection model, mice (9–17 per group) were intraperitoneally injected with XAB06 (12.5, 25, or 50 mg/kg of body weight), non-immune control IgG (50 mg/kg), or Gremubamab (1 mg/kg) 3 h before challenge with the PAO1 strain (LD100, in 5% porcine gastric mucin). Mice were then monitored at 1, 4, 8, 12, 18, and 24 h after infection and twice daily for subsequent days until the end of the 10-day observation period. A clinical scoring, including physiological and behavioral parameters, was designed based on reference 41 with some modifications (Table 1). Analgesic treatment with buprenorphine (Virbac, Carros, France) was provided by subcutaneous injection (0.1 mg/kg of body weight, three times/day) and in the drinking water (0.009 mg/mL) during the first 48 h of the experiment.

**TABLE 1:** Clinical scoring of mice

		Score
Coat condition	Smooth	0
	Slight piloerection	1
	Moderate piloerection	2
	Marked piloerection	3
Activity	Normal	0
	Hunched	1
	Reduced interaction with animals	2
	Marked reduced interaction with animals	3
	Passive behavior, tremors	4
Palpebral aperture	Open	0
	slightly tightened	1
	Half closed	2
	Sunken and half closed	3

## RESULTS

### Immunization of double KO pigs with a monovalent or a pentavalent PA preparation

Although 20 serotypes of PA have been described, some of them are more frequently encountered in clinical situations (Table 2). This is the case for serotype O6, which represents around 20% of blood, pneumonia, or urinary tract infections. The combination of O1, O2, O4, O6, and O11 serotypes accounts for more than 60% of infections (31–34). Thus, targeting these specific serotypes could potentially confer protection against most of the invasive PA isolates. We compared the feasibility of immunizing double KO pigs with a whole inactivated monovalent (ATCC 33354, O6 serotype) or a pentavalent (ATCC 33348, ATCC 33349, ATCC 33351, ATCC 33354, and ATCC 33358, i.e., O1 + O2 + O4 + O6 + O11 serotypes) PA preparation. Sera were collected after four immunizations, and their reactivity (binding and C1q recruitment) was evaluated in a whole-bacterium ELISA assay against the O6 serotype, a mixture of O1 + O2 + O4 + O6 + O11 serotypes, or O1, O2, O3, O4, O5, O6, O9, O10, and O11 serotypes individually (Fig. 1A).

**TABLE 2:** Distribution of O serotypes in clinical PA isolates^*a*^

	Nasrin et al. (31)	Lu et al. (34)	Recio R et al. (33)	Barrio-Tofino et al. (32)
	Blood/ cerebrospinal fluid	Pneumonia	Blood	Pneumonia/Urinary track infectionI/soft tissues/blood
	*N* = 413 (North America, Europe, Africa, Asia)	*N* = 143 (Europe)	*N* = 243 (Spain)	*N* = 1445 (Spain)
Serotype	Prevalence (%)			
**O1^*b*^**	**15**	**8**	**16**	**15**
**O2**	**7**	**9**		**4**
O3	7	4		7
**O4**	**1**	**2.4**	**21**	**11**
O5	4			5
**O6**	**22**	**29**	**16**	**18**
O7	4	2.4		3
O8		0.8		1
O9	4	1.6		1
O10	1	10		3
**O11**	**22**	**23**	**15**	**13**
O12	1	0.8		1
O15	1	0.8		1
NT	5	8		14.5
**O1 + O2+O4+O6+O11**	**67**	**69**	**68**	**61**

^*a*^ Results from four studies are presented in the table. Type of infection, number of isolates, and geographic localization are mentioned. NT, non-typeable.

^*b*^ Bold indicates PA serotypes in the pentavalent preparation.

**Fig 1:**
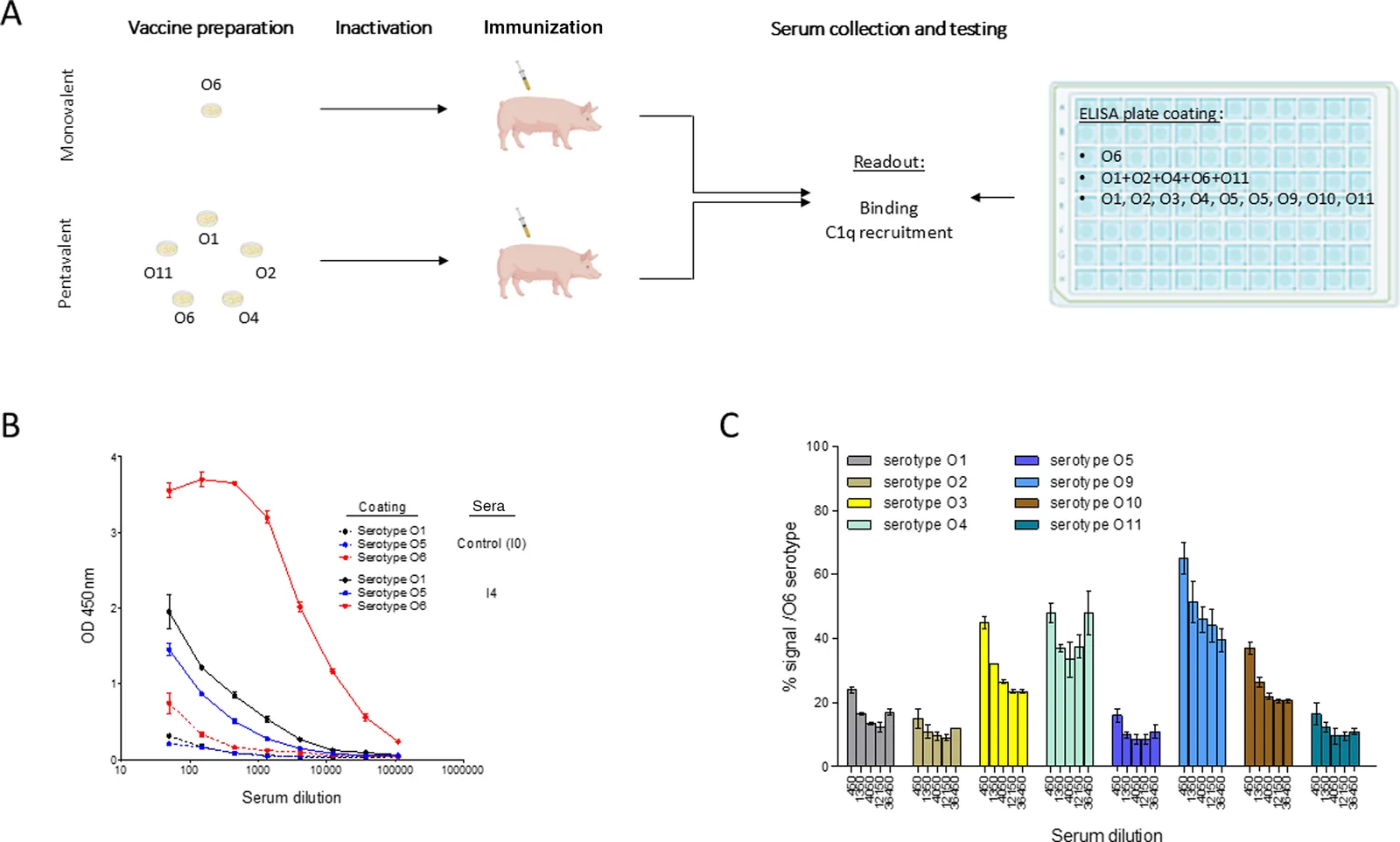
Production of anti-PA GH-pAb. (**A**) The program of immunization with monovalent or pentavalent vaccine and the tests performed to characterize the sera generated are depicted. Animals were immunized every 2 weeks. Serum was from the fourth immunization and tested for anti-PA activity. (**B**) Binding of monovalent serum (1/50, then 1:3 serial dilution) before immunization (I0, control serum) or after four immunizations (I4) on O6, O5, and O1 PA serotypes was investigated by ELISA. One of two experiments is shown. (**C**) Cross-reactivity of monovalent serum against each PA serotype individually was investigated and expressed as the percentage of signal obtained against the O6 serotype (*n* = 2).

We first assessed the reactivity of the monovalent serum. Strong binding was observed against the O6 serotype bacteria used for the immunizations, with a plateau reached at 1/450 dilution and a positive signal maintained until dilution >1/100,000 (Fig. 1B). Cross-reactivity was investigated against O1 and O5 PA serotypes. Although both PA serotypes were recognized, binding was much lower compared to binding to the O6 serotype. We then decided to methodically measure the cross-reactivity of monovalent serum against O1, O2, O3, O4, O5, O9, O10, and O11 PA serotypes. ELISA plates were coated with each PA serotype and with O6 serotype as an internal calibrator. For each serotype, binding was then expressed as the percentage of signal obtained with O6 serotype at the tested dilution. Weak-to-moderate cross-reactivity was observed for serotypes O1, O2, O5, and O11 (<20% of serotype O6 signal) and O3, O4, O9, and O10 (20%–50% of serotype O6 signal), respectively (Fig. 1C).

Overall, cross-reactivity of monovalent serum against PA serotypes was limited. To broaden the reactivity of pig IgG toward various PA serotypes, a pentavalent vaccine, comprising the five PA serotypes frequently encountered in clinical settings, was prepared and used for pig immunization. Pentavalent and monovalent sera were then compared in whole-cell ELISA against the O6 serotype, a mixture of O1, O2, O4, O6, and O11 serotypes, or each PA serotype independently. Reactivity of pentavalent serum against the O6 serotype was comparable to monovalent serum reactivity (Fig. 2A). By contrast, pentavalent serum showed much stronger binding to the pentavalent preparation than to the monovalent serum (Fig. 2B). We then compared the binding of monovalent and pentavalent sera against eight individual PA serotypes. Except for serotype 9, pentavalent serum displayed superior binding to all the serotypes tested individually (Fig. 2C). The gain in binding was evaluated for each serotype. It was about 1.5-fold for serotype O3, 2-fold for serotypes O2, O4, and O10, 3-fold for serotype O5, and 5-fold for serotypes O1 and O11 (Fig. S1).

**Fig 2:**
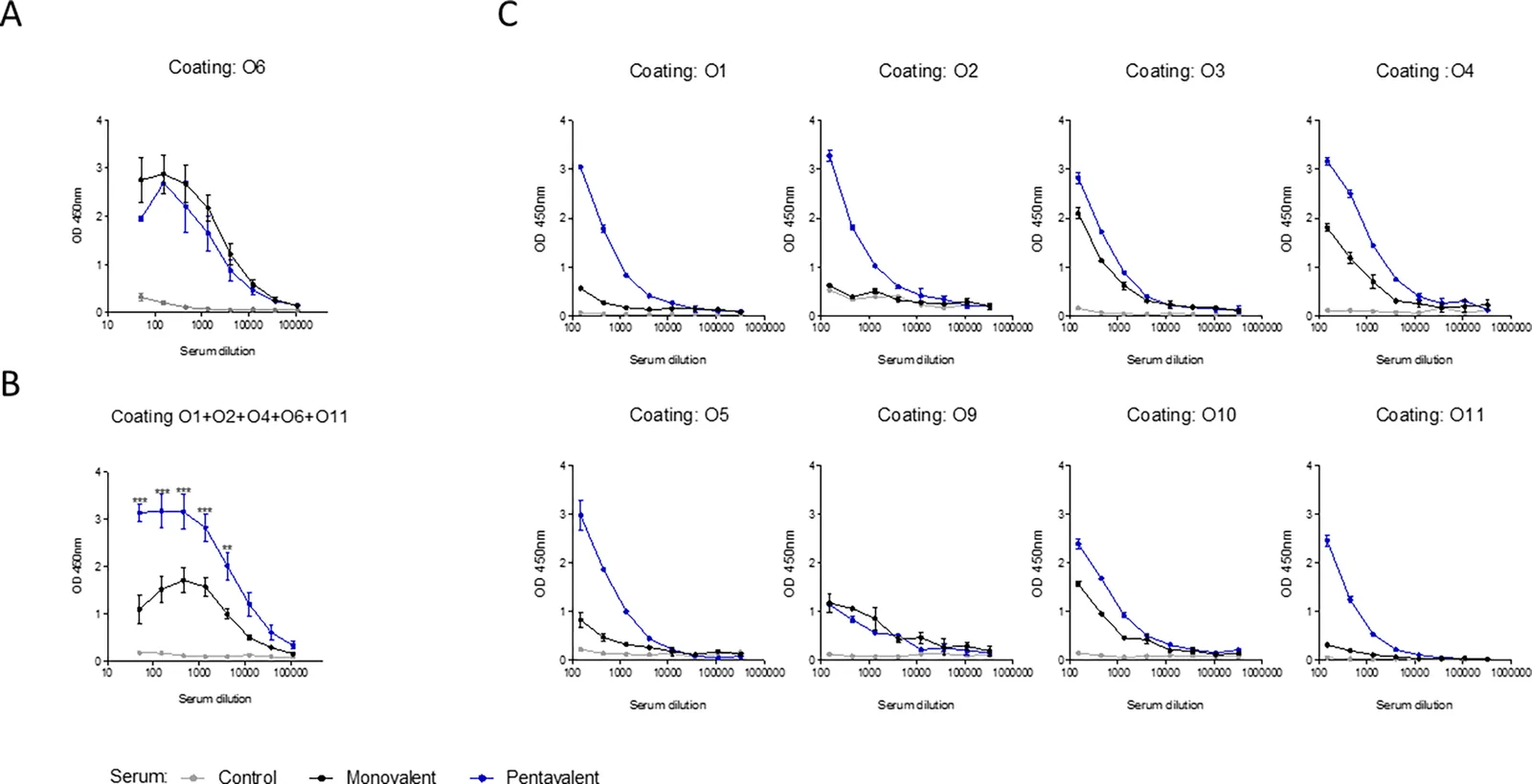
Comparison of binding activity between monovalent and pentavalent sera. Monovalent, pentavalent, or control non-immunized (CT) sera were compared for their binding against O6 serotype (**A**) or a mixture of O1 + O2 + O4 + O6 + O11 serotypes (**B**). Serum dilution: 1/50, then 1:3 serial dilution. Data are expressed as mean ± standard error of the mean (*n* = 3). Two-way ANOVA with Bonferroni *post hoc* test was performed. **P < 0.01; ***P < 0.001. (**C**) Sera were tested against each serotype individually. Serum dilution: 1/150 (**C**), then 1:3 serial dilution. One of two experiments is shown.

IgG from pentavalent serum (referred to below as XAB06) was then purified by protein A chromatography. Reactivity of XAB06 against O6 serotype PA was confirmed by ELISA and immunofluorescence staining (Fig. 3A and B), while slight reactivity against other bacterial species (*Acinetobacter baumannii*, *Escherichia coli*, *Klebsiella pneumoniae,* or *Enterobacter cloacae*) was observed (Fig. 3A). Activation of the complement system is an important downstream effector function after Ab binding (42). We thus assessed the ability of XAB06 to recruit C1q after binding to PA. Human C1q engagement was observed for all the serotypes tested after recognition by XAB06 (Fig. 4). Opsonophagocytic killing assays revealed moderate lysis of *Pseudomonas* after XAB06 treatment (30%) (not shown), suggesting that this is not the main mechanism of action. Besides, we evaluated the reactivity of XAB06 against human blood cells. Unresponsiveness of XAB06 against peripheral blood mononuclear cells was demonstrated by the absence of binding (Fig. 5A), complement-mediated lysis (Fig. 5B), or cytokine secretion (Fig. 5C).

**Fig 3:**
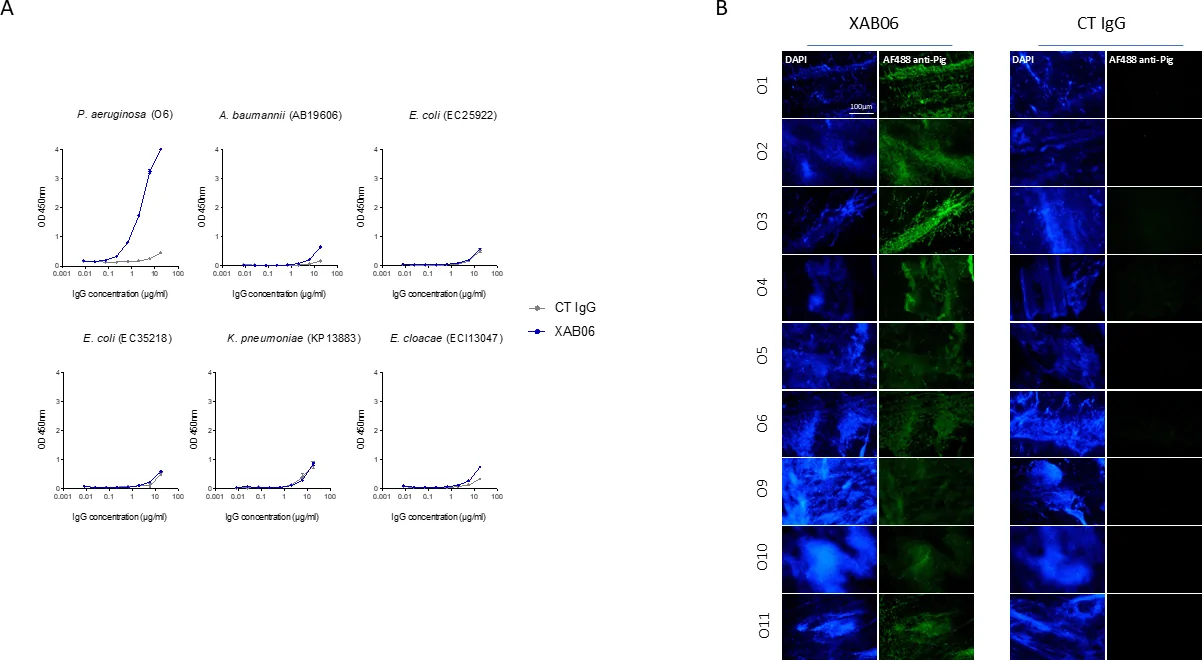
Investigation of XAB06 binding activity. (**A**) XAB06 or non-immune IgG (CT IgG) binding activity against PA and unrelated bacterial strains (*E. coli, A. baumannii, K. pneumoniae,* or *E. cloacae*) was evaluated by ELISA. IgG concentration: 18 µg/mL, followed by 1:3 serial dilution. One of two experiments is shown. (**B**) Binding of XAB06 against PA serotype individually was investigated by fluorescence microscopy (40×; exposure time, 100 ms).

**Fig 4:**
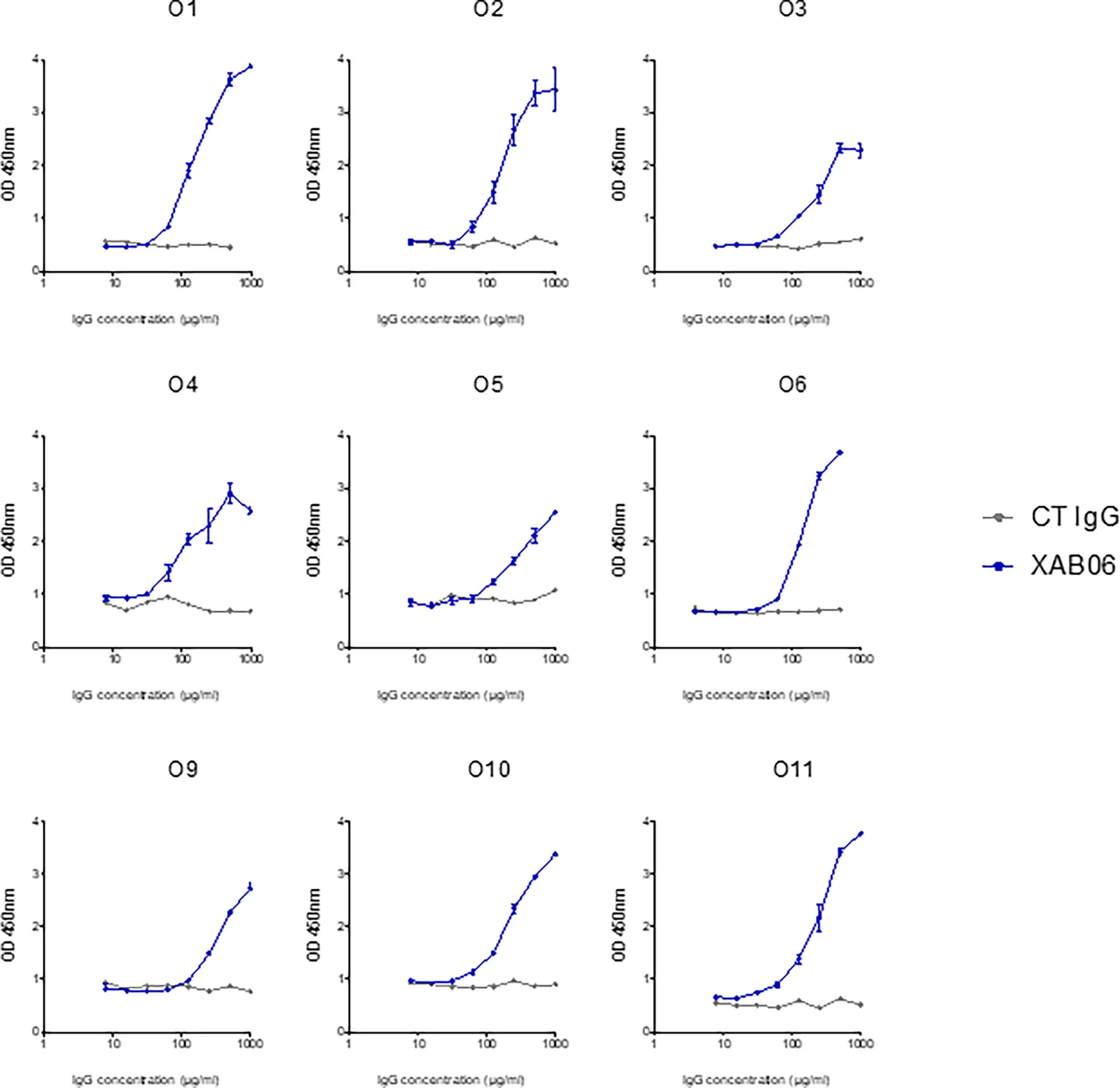
C1q recruitment assay. The capacity of XAB06 to recruit human C1q after binding to PA serotypes O1, O2, O3, O4, O5, O6, O9, O10, or O11 was investigated by ELISA. An IgG from a non-immunized animal was used as a control (CT IgG). IgG concentration: 1,000 µg/mL, followed by 1:3 serial dilution. One of two experiments is shown.

**Fig 5:**
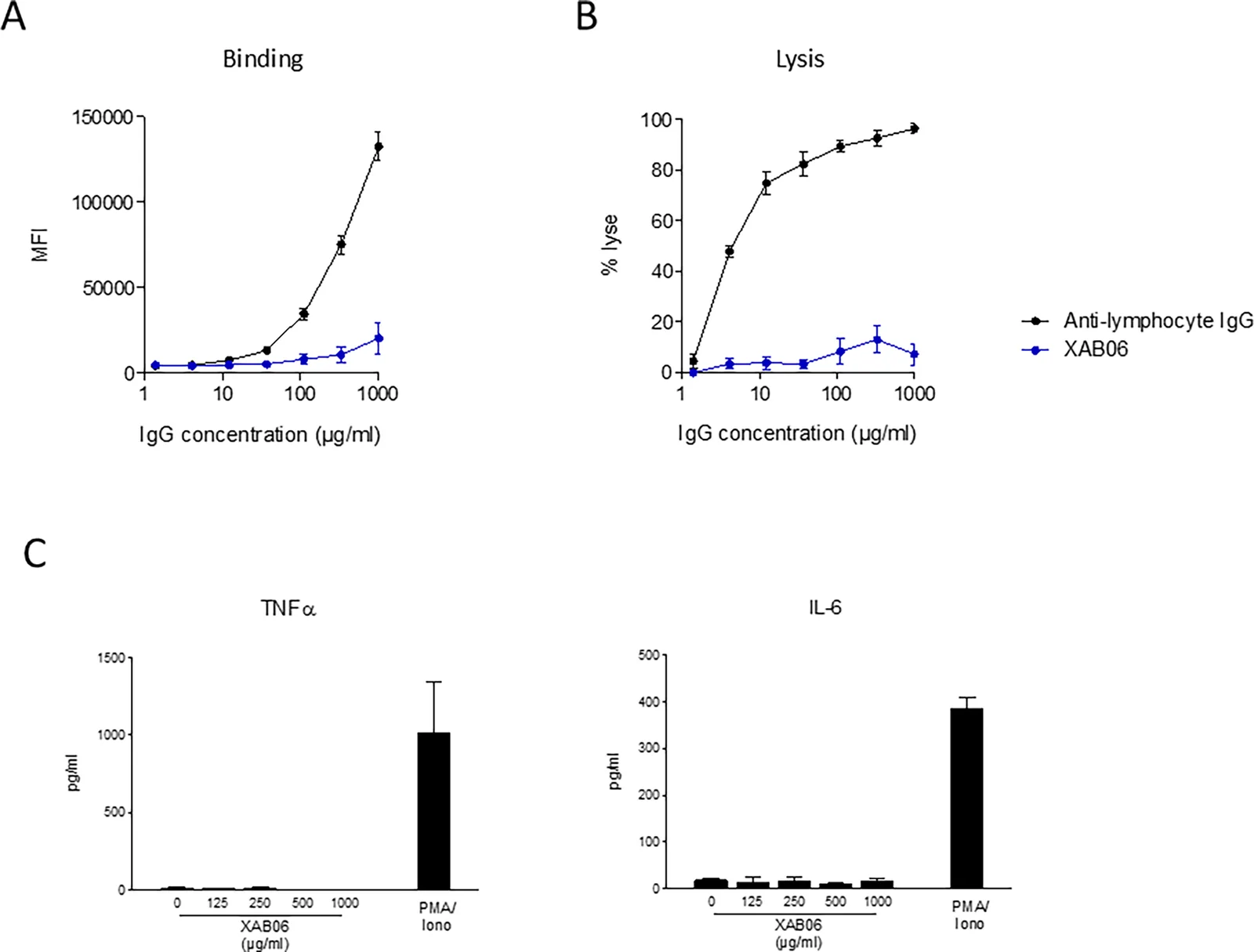
XAB06 shows no reactivity against human blood cells. Binding (**A**) and complement-dependent cytotoxicity (**B**) against human peripheral mononuclear cells were investigated. An anti-lymphocyte pig IgG was used as a positive control (*n* = 4). (**C**) Investigation of IL-6 and TNFα secretion by human PBMCs exposed to XAB06. PMA and Ionomycin were used as positive controls (*n* = 3).

### Comparison of XAB06 with Gremubamab

XAB06 was then compared with the bispecific mAb Gremubamab, which recognizes the type 3 secretion system component PcrV and Psl exopolysaccharide. PcrV binding was much stronger with Gremubamab (Fig. 6A), suggesting that PcrV is not a major target of XAB06. Yet, whole-bacterium ELISA showed that XAB06 recognized the nine PA serotypes tested more efficiently (Fig. 6B).

**Fig 6:**
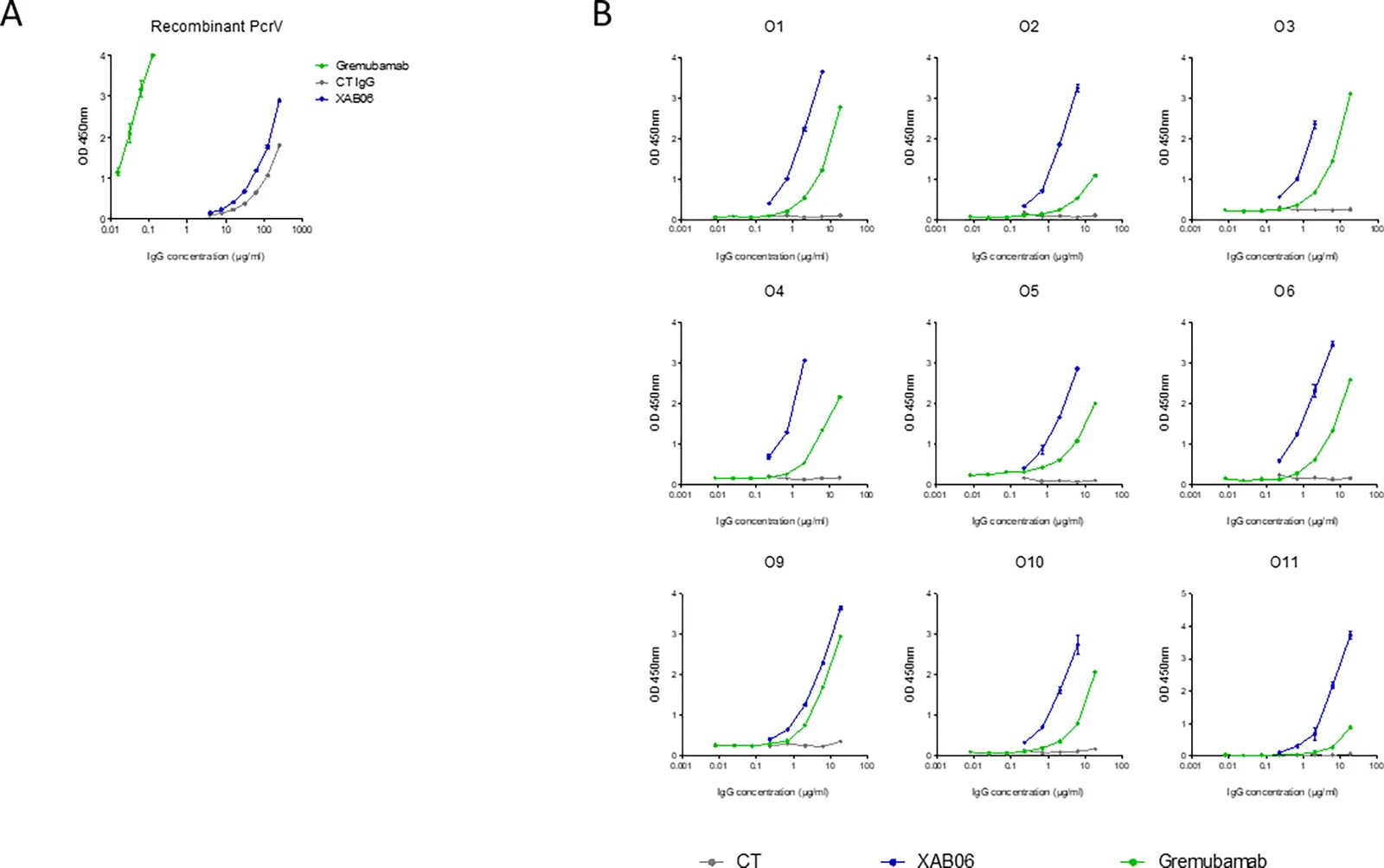
Comparison of XAB06 and Gremubamab. Binding of XAB06 and Gremubamab to recombinant PcrV (**A**) or whole bacteria (**B**) was investigated by ELISA. HRP-conjugated protein A was used as a common detection system for human (Gremubamab) and swine (XAB06) IgG. IgG concentration: 18 µg/mL, followed by 1:3 serial dilution. One of two experiments is shown.

### XAB06 reduced PA motility, biofilm synthesis, and pyocyanin production

We then investigated the ability of XAB06 to impact the biological activity of PA on the ATCC 33354 (O6 serotype) strain and the reference strain PAO1. Binding of XAB06 on PAO1 was confirmed in Fig. 7A. Both strains were then tested for motility on semi-solid agar plates and for biofilm and pyocyanin production. XAB06 dramatically reduced swarming motility of PAO1 and, to a lesser extent, motility of ATCC 33354 (Fig. 7B). Notably, inhibition appears stronger with the strain that exhibited lower swarming potential. XAB06 affected biofilm synthesis of PA ATCC 33354 (42% reduction at 500 µg/mL) and PAO1 (55%, 65%, and 72% drop at 125, 250, and 500 µg/mL, respectively) (Fig. 7C). Finally, XAB06 dramatically decreased the production of pyocyanin by PAO1 strain (55%, 76%, and 82% at 125, 250, and 500 µg/mL, respectively, Fig. 7D). We did not detect substantial pyocyanin production by PA ATCC 33354 strains.

**Fig 7:**
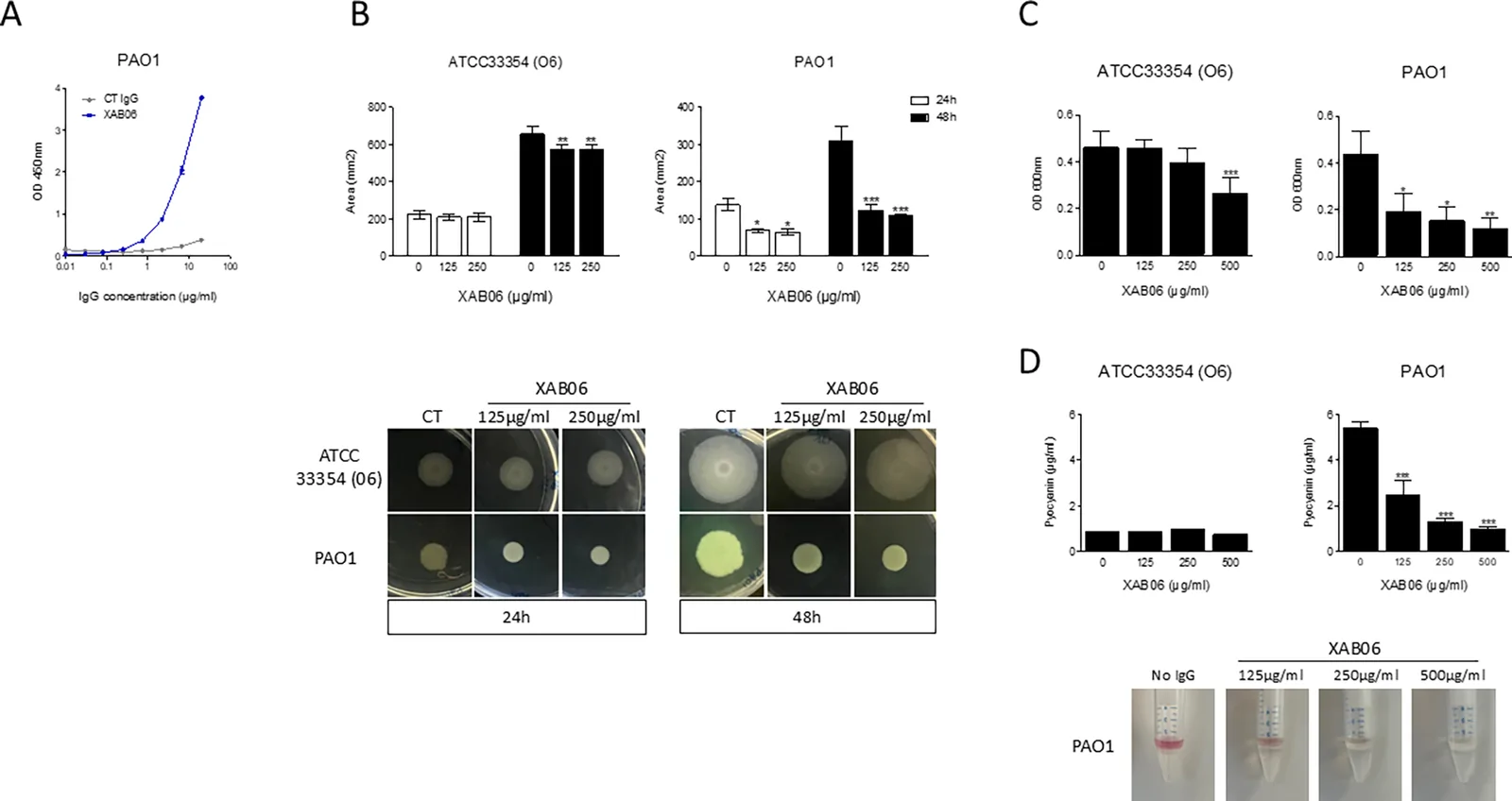
XAB06 antibacterial *in vitro* activity. Activity of XAB06 was investigated against ATCC 33354 (serotype O6) and PAO1 strains. (**A**) Binding of XAB06 on the PAO1 strain was investigated by whole-cell ELISA. IgG concentration: 18 µg/mL, followed by 1:3 serial dilution. One of two experiments is shown. Bacterial motility (**B**) was assessed on semi-solid agar plates. Measurement of colony area is shown in the top, and images of the colonies are shown in the bottom. Biofilm production (**C**) was determined by crystal violet staining. Determination of pyocyanin production (**D**). Quantification after chloroform extraction (top) and an image of the coloration for the PAO1 strain (bottom) are shown. Data are expressed as mean ± SEM (*n* = 3). Two-way ANOVA with Bonferroni *post hoc* test. *P < 0.05; **P < 0.01; and ***P < 0.001.

### Antibacterial activity of pentavalent IgG in a mouse sepsis model

We finally evaluated the antibacterial activity of XAB06 *in vivo* in a mouse sepsis model. Mice were treated with increasing concentrations of XAB06 (12.5, 25, or 50 mg/kg) or with Gremubamab (1 mg/kg), 3 h before a lethal challenge with the PAO1 reference strain (Fig. 8A). Non-immune IgG was used as a control. Health condition and survival were subsequently monitored for 10 days. XAB06 treatment significantly reduced the clinical symptoms of sepsis (*P* < 0.01 and *P* < 0.005 at 12 and 18 h, respectively) and protected mice in a dose-dependent manner (*P* < 0.0001) (Fig. 8B). Gremubamab’s impact on clinical score and mouse survival was limited. The antibacterial activity of XAB06 was then investigated on two supplementary PA strains: the PA103 reference strain and H27244 (ST235 high-risk clone). Both strains were recognized by XAB06 (Fig. S2), and protection was confirmed after XAB06 treatment (Fig. 8C).

**Fig 8:**
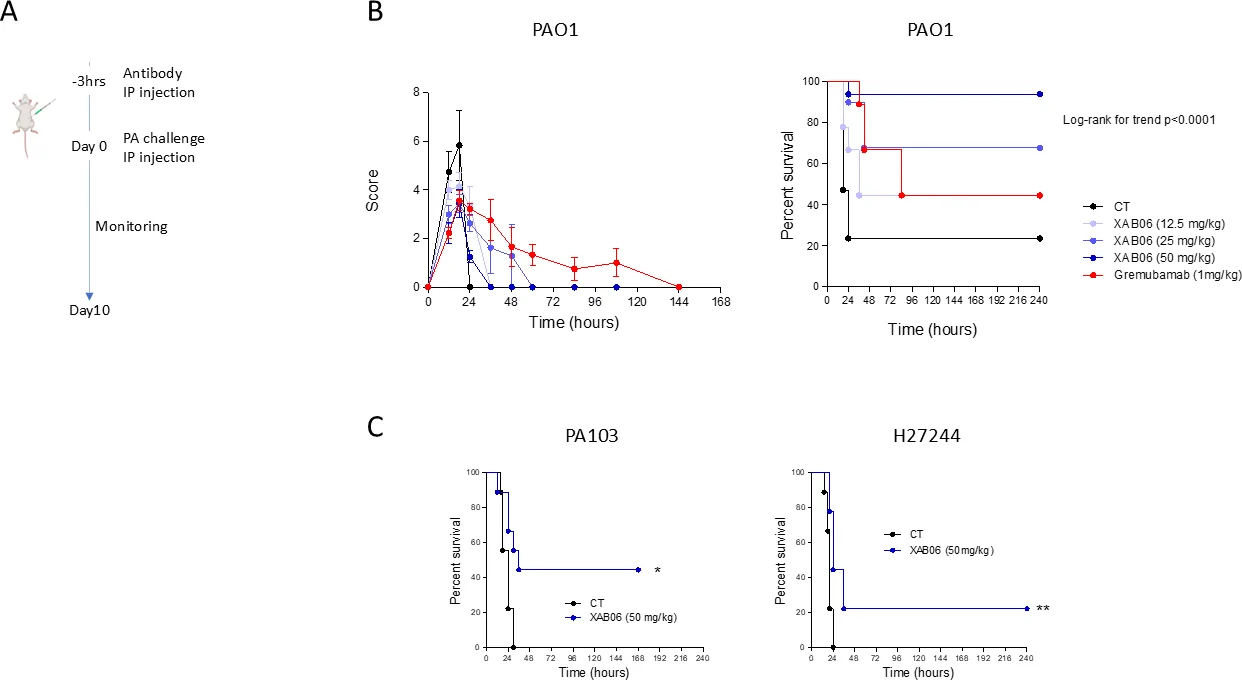
XAB06 protects from PA challenge in a mouse sepsis model**.** (**A**) Presentation of the sepsis model. Mice were injected intraperitoneally with IgG 3 h before a lethal challenge with the PA (about 1.4 × 10^4^ CFU/mouse). (**B**) Sepsis model with the PAO1 strain. Mice were treated with increasing concentrations of XAB06 (12.5, 25, and 50 mg/kg). Gremubamab (1 mg/kg) was used as a control. Clinical score (left panel); data are expressed as mean ± SEM. Kaplan-Meier curves of mouse survival are shown in the right panel. (**C**) Sepsis model with PA103 or H27244 (high-risk ST235 clone). Kaplan-Meier curves of mouse survival are shown.

## DISCUSSION

There is an urgent need to design innovative therapies against PA, particularly MDR and extensively drug-resistant strains. Among emerging therapeutic approaches, GH-pAbs offer an attractive strategy by their capacity to recognize multiple antigens on bacterial targets. We have already demonstrated both the potential and safety of GH-pAb in various preclinical or clinical contexts, including infectious diseases (25, 43), transplantation (29), or cancer (44, 45).

Twenty PA serotypes have been described, with considerable variations in surface polysaccharides and other virulence-associated components (30). To achieve broad protection against PA, it is necessary to address its antigenic diversity. Within this scope, whole-cell immunization is a compelling approach (46–48). Yet, it usually necessitates pathogen inactivation through chemical or physical means (49–51). As we wanted the broadest possible activity, heating was preferred over chemical treatments to avoid washes and retain any soluble factors in the vaccine preparation. We observed limited cross-reactivity against PA serotypes with the monovalent preparation. Whereas this suggests LPS as an important/majority target of the monovalent preparation, it also evidences the presence of shared antigens between PA serotypes. We thus decided to combine five PA serotypes for vaccine preparation based on their frequency of distribution in clinical settings (31, 34) and/or their association with MDR profile (serotypes O4 and O11) or pathogenicity (32, 33). Overall, XAB06, the resulting pentavalent IgG preparation, displayed broad reactivity against all the serotypes/strains tested, and no loss of activity against the O6 serotype compared to the monovalent preparation. This suggests an absence of immune interference with the pentavalent vaccination. Importantly, XAB06 retained binding to the well-characterized reference strain PAO1, which was not included in the immunization mixture, or to the O12 serotype, associated with MDR (not shown).

Comparative studies with the bispecific Gremubamab mAb targeting PcrV and the PSL exopolysaccharide (21) were performed. Although Gremubamab showed undeniable superiority in binding to recombinant PcrV, whole-cell ELISA showed a stronger binding profile of XAB06 on whole bacteria, presumably through recognition of multiple bacterial structures, including surface polysaccharides, outer membrane proteins, and virulence factors. Notably, XAB06 was specific for PA and displayed very low reactivity against the unrelated gram-negative bacteria tested in this study, thereby minimizing the risk of off-target effects and damage to the microbiota. In addition, no reactivity with human blood cells was noticed, further supporting a favorable safety profile.

Beyond binding, XAB06 displayed biological activity against PA virulence factors. Bacterial swarming motility was inhibited, suggesting that XAB06 impaired the production of rhamnolipids (52, 53). Furthermore, biofilm formation and pyocyanin production were markedly impaired by antibody treatment. Higher doses of XAB06 were required in these biological assays (IC50, 61.6 and 94.7 µg/mL for the biofilm and the pyocyanin assays, respectively) compared to the binding. This likely reflects differences in assay format, with functional assays using growing bacteria, and in the readout measured (recognition versus antibacterial activity). Together, these findings suggest that XAB06 targets soluble PA secreted factors and interferes with several interconnected virulence networks, possibly through disruption of quorum-sensing signaling process (10, 54). The simultaneous targeting of multiple determinants would render the emergence of resistance against XAB06 more difficult than with single-target agents such as monoclonal antibodies or antibiotics. Moreover, interfering with virulence factors is supposed to apply less selective pressure for the development of bacterial resistance than traditional antibiotics (55, 56).

The protective potential of XAB06 was first evaluated in a murine sepsis model using the PAO1 strain. Remarkably, passive immunization with XAB06 reduced the sepsis symptoms and conferred protection in a dose-dependent manner, even though PAO1 (serotype O5) was not one of the serotypes used in the initial immunization of pigs. Protection was confirmed, although to a lesser extent, against the PA103 reference strain and H7244 strain (ST235 high-risk clone). Considering a total blood volume of 8% of the body weight and a plasmatic distribution, expected blood concentrations of XAB06 would be 156, 312.5, and 625 µg/mL following injections of 12.5, 25, and 50 mg/kg. This suggests an active blood concentration of XAB06 within the range of the active concentration in the functional assays *in vitro*. Besides, according to the Food and Drug Administration guidelines for conversion between animal and human doses based on body surface area scaling factor (57), the dose we used in mice (50 mg/kg) equates to 4 mg/kg in humans, within the classical ranges of pAb doses administered in humans (29, 58).

In this proof-of-concept study, we used a standard prophylactic acute intraperitoneal model. Although largely described (59, 60), this model has limitations, and further investigations will be useful to evaluate XAB06 activity in therapeutic situations (with treatment administered after bacterial challenge) or in organ-/tissue-specific settings using pneumonia or urinary tract infection models. Furthermore, today, no truly appropriate control currently exists, as no therapeutic polyclonal antibody against PA has yet been developed. Serum from IC43-vaccinated patients could be a polyclonal alternative but was not available to us. We thus used Gremubamab, a bispecific antibody, as a comparator for XAB06. Assessing the activity of XAB06 against other PA strains or clinical isolates, determining the targets of XAB06, and determining the molecular basis of its activity will be useful to precisely gauge the therapeutic capacity of XAB06.

The therapeutic potential of antibodies in the fight against bacteria is increasingly recognized (13, 24, 61). Antibacterial antibodies currently approved, such as Raxibacumab (*Bacillus anthracis*) or Bezlotoxumab (*Clostridium difficile*), primarily target bacterial toxins (62). The evaluation of Gremubamab therapeutic activity is still ongoing. In this context, XAB06 could be envisaged for therapeutic use either as a standalone antibacterial agent, owing to its direct neutralizing and anti-virulence effects.

In summary, XAB06 represents a novel therapeutic option for combating PA. By simultaneously targeting multiple epitopes and virulence pathways, it combines broad-spectrum recognition, potent biological activity, and high specificity. Together with the already demonstrated safety and tolerability in humans of GH-pAbs (25, 29), these features make XAB06 a promising candidate for future therapeutic development against PA infections.

## SUPPLEMENTAL MATERIAL

Supplemental figures aac.00277-26-s0001.docx Fig. S1 and S2.

ASM does not own the copyrights to Supplemental Material that may be linked to, or accessed through, an article. The authors have granted ASM a non-exclusive, world-wide license to publish the Supplemental Material files. Please contact the corresponding author directly for reuse.
