# Supplementary material for: XAB06, a glyco-humanized broad-spectrum anti-Pseudomonas aeruginosa polyclonal antibody, protects mice in a lethal sepsis model
Source: Antimicrob Agents Chemother. 2026 Sep 3;70(10):e00277-26. doi: 10.1128/aac.00277-26 (PMC13689433; doi:10.1128/aac.00277-26)
Supplement: Supplementary file 1 [file aac.00277-26-s0001.docx]

**Supplementary Figure 1. Quantitative comparison between the pentavalent and the monovalent serums.** Each ELISA plates were coated with the O6 serotype (the calibrator) and the PA serotype investigated. Binding of monovalent or pentavalent serum was then determined and then expressed as the percentage of the maximum binding (the binding of an anti-O6 monovalent preparation against a O6 coating).

**Supplementary Figure 2. Binding of XAB06 to PA103 strain and ST235 high risk clone.** ELISA plates were coated with whole bacteria. XAB06 or CT IgG (18µg/ml then 1:3 serial dilution) were added to the plates. Detection was performed with HRP-conjugated protein A.
